# A meta-analysis of the impact of COVID-19 on liver dysfunction

**DOI:** 10.1186/s40001-020-00454-x

**Published:** 2020-11-04

**Authors:** Zeng-hong Wu, Dong‑liang Yang

**Affiliations:** Department of Infectious Diseases, Union Hospital, Tongji Medical College, Huazhong University of Science and Technology, Wuhan, 430022 China

**Keywords:** COVID-19, SARS-CoV-2, Liver injury, Liver dysfunction, Meta-analysis

## Abstract

**Background:**

The novel coronavirus disease 2019 (COVID-19), which is caused by the severe acute respiratory syndrome coronavirus 2 (SARS-CoV-2) is leading to a worldwide pandemic. Except representative manifestation of pneumonia and acute respiratory symptoms, COVID-19 patients have also shown different levels of liver injury or liver dysfunction. The aim of our study was to explore the probable clinical severity and mortality of COVID-19 patients and their liver dysfunction.

**Method:**

A combination of computer and manual retrieval was used to search in Medline through PubMed, EMBASE and Web of Science. Review Manager 5.3 software was used to examine the heterogeneity among the studies and to calculate the combined effect value (OR, 95CI). Subgroup analysis, sensitivity analysis, and publication bias test were also performed.

**Results:**

We found a significant connection between liver dysfunction and mortality of COVID-19 patients with a pooled OR of 1.98 (95% CI 1.39–2.82; *P* = 0.0002). There was a significant association between AST and severity of COVID-19 with a pooled OR of 4.48 (95% CI 3.24–7.21; *P* < 0.001), and a pooled WMD of 3.35 (95% CI, 2.07 to 4.64; *P* < 0.001). In addition, there was a significant difference between TBIL and severity of COVID-19, with a pooled OR of 1.91 (95% CI 1.40–2.60; *P* < 0.001), and with a pooled WMD of 1.18 (95% CI, 0.78 to 1.58; *P* < 0.001)**.**

**Conclusion:**

The mortality and severity of COVID-19 patients are significantly associated with liver dysfunction. The non-survivors and severe COVID-19 patients have elevated serum AST levels than the survivors and non-severe COVID-19 patients. The results of this study form a basis for better clinical liver management of patients with COVID-19.

## Background

Coronavirus (CoV) is an enveloped positive single-stranded RNA virus. It is widely distributed in humans and animals. It is known to cause human respiratory infections [1]. The ongoing COVID-19 pandemic caused by the new severe acute respiratory syndrome coronavirus 2 (SARS-CoV-2) is rapidly developing [2]. COVID-19 is caused by a virus, SARS-COV-2, and its major symptoms are fever, cough, and dyspnea, and minor symptoms are alteration of the smell and taste, gastrointestinal symptoms, headache, and cutaneous manifestations [3–5]. SARS-CoV-2 enters cells through the angiotensin converting enzyme 2 (ACE2) which is its intended receptor [6]. Currently, there are no specific/targeted drugs or vaccines for SARS-CoV-2. In many parts of the world, the number of SARS-CoV-2 positive patients are increasing exponentially. As of 18th April, 2020, there were 2,251,633 positive cases and 154,329 deaths worldwide. This suggested that the total death rate of COVID-19 was 6.85%. Clinical symptoms of COVID-19 are pneumonia and acute respiratory symptoms. However, COVID-19 patients also have varying levels of liver injuries or liver dysfunction though the results are controversial. Yang et al*.* reported that there were no differences in liver function among survivors and non-survivors of COVID-19 [7]. A recent study reported that 318 out of the 417 COVID-19 patients tested during hospitalization had aberrant liver test results while 90 patients had liver injury. The use of ritonavir/lopinavir also increased the occurrence of liver injury [8]. Chen et al*.* reported that there were liver enzyme abnormalities in 99 COVID-19 patients in Wuhan. Among the 99, 43 (43.4%) had elevated aspartate aminotransferase (AST) and serum alanine aminotransferase (ALT) [9]. However, in a cohort of 548 COVID-19 patients from mainland China, there were no significant differences in ALT levels between non-severe (*n* = 279) and severe (*n* = 269) patients (*P* = 0.683) [10]. Similarly, Wan et al*.* retrospectively studied 135 hospitalized COVID-19 patients and reported that there were no significant differences in ALT (*P* = 0.73) and total bilirubin (TBIL, *P* = 0.07) between mild cases (*n* = 95) and severe cases (*n* = 40) [11]. These contradicting reports have now attracted widespread attention. As such, this meta-analysis aimed to explore the probable clinical severity and mortality of COVID-19 patients and their liver dysfunction. The study further analyzed the likelihood of COVID-19 patients to have bad liver injuries. The results of this study will help researchers and clinicians in formulating stronger prevention guidelines as well as have effective responses to COVID-19. Ultimately, this will contribute to effective management and treatment of liver injuries.

## Methods

### Search summary

Studies involving SARS-CoV-2 or COVID-19 or liver injury/dysfunction were included. In order to find relevant original articles, we conducted a comprehensive search in the database, involving Medline through PubMed, EMBASE and Web of Science, and using the following words: "COVID-19", "SARS-CoV-2", "Wuhan virus", "Chinese virus", "novel coronavirus 2019", "2019 nCoV", "Wuhan coronavirus", ``2019 Coronavirus", "Characteristics", "Characteristics" and "Liver". As of April 17, 2020, the papers have been searched in the language range. We also refer to the recognized literature to find other qualified research subjects. We first screened the article title and abstract, as well as publications that may involve data on COVID-19 or SARS-CoV-2 and liver injury/dysfunction.

### Inclusion and exclusion standard

This study had no national restrictions. All studies reporting on COVID-19 non-survivors/survivors, non-severe/severe and laboratory confirmed COVID-19 patient data were included in the study. Moreover, the studies had to be limited to humans, include raw data, be published in English and be in either abstract form or full text. Repeated studies, letters, case reports, abstracts, and comments were excluded from the study. Data collected from each study included the year of publication, name of first author, sample size, and age of COVID-19 patients.

### Statistical analysis

The quality of each study was evaluated using the Newcastle–Ottawa scale while the meta-analysis was conducted through the Review Manager 5.3 software. Heterogeneity was evaluated by calculating the *I*^2^ index. *I*^2^values less than 25%, 25–50%, 50–75% and 75–100% were homogeneous, or had low, medium, and high heterogeneity levels, respectively. The random effect model (REM) was applied if the *I*^2^ value is > 50% while the fixed effect model (FEM) was applied if the *I*^2^ value is < 50%. The combined odds ratio (OR) and weighted mean difference (WMD) of different studies with corresponding 95% confidence intervals (CI) were used to assess the relationship between liver dysfunction and COVID-19. The sensitivity analysis was repeated to evaluate the impact of each study in the analysis by subsequently excluding different individual studies each time. Based on the method of Hozo et al. [12], the mean and standard deviation were inferred from the sample size, median and interquartile range (IQR) when the continuous variables were not available.

## Results

### Study processing

**Fig. 1:**
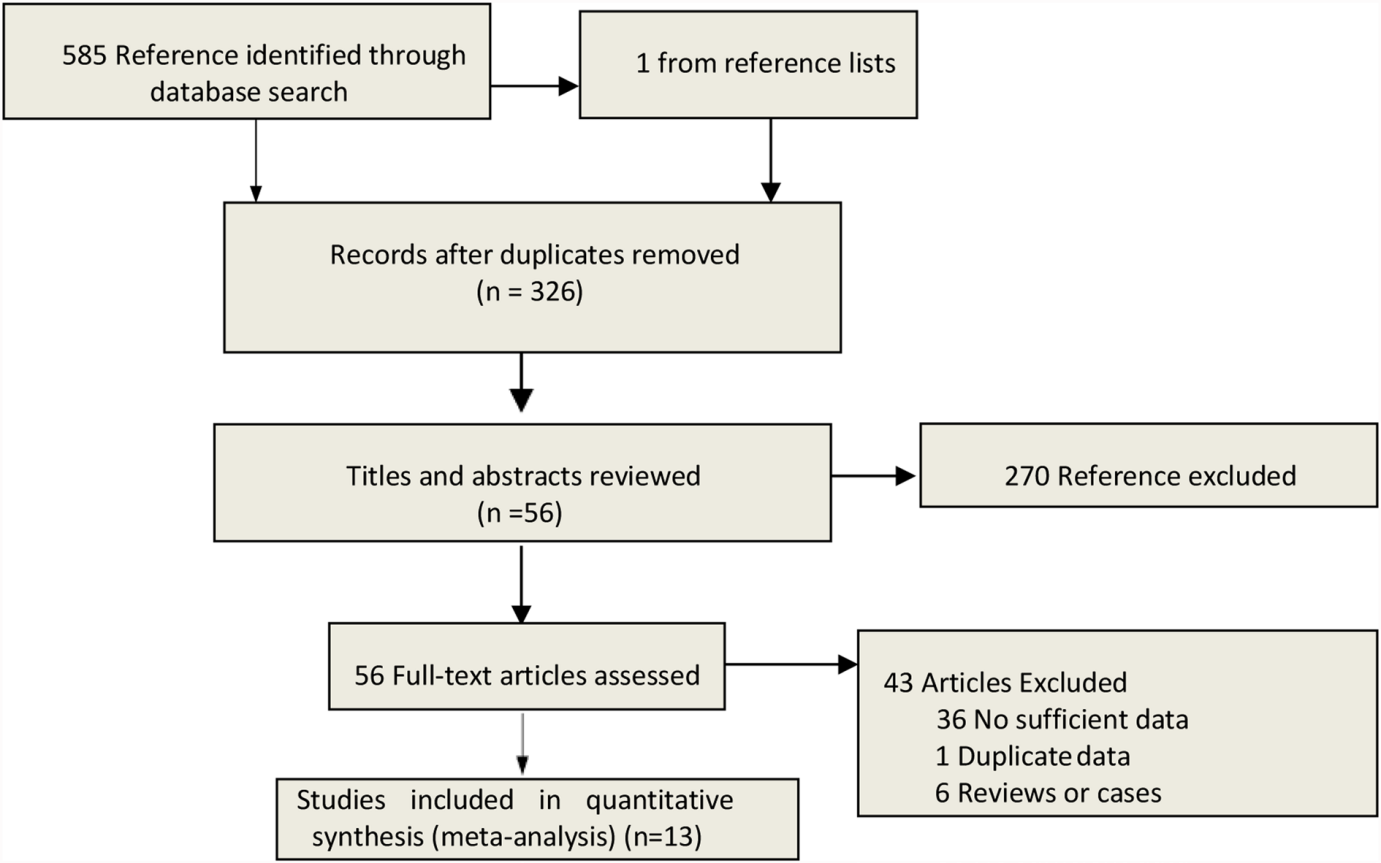
Search strategy to identify articles on the relationship between liver dysfunction and COVID-19

Five hundred and eighty-five relevant articles and one from the reference list were identified. Exclusion of duplicates brought down the number to 326 studies. The 326 studies were further screened if they meet the inclusion criteria. This resulted in the exclusion of 270 studies. Forty-three more studies were excluded after scanning of the entire body text of the 56 remaining studies. The remaining 13 articles meet the eligibility criteria [7, 8, 10, 11, 13–21]. The steps of document retrieval are shown in Fig. 1. Similarly, the study characteristics are shown in Tables 1 and 2. The meta-analysis involved 3,722 cases (285 dead vs 748 alive; 1968 non-severe vs 721 severe). All studies were conducted and published in 2020. One of the studies (Reference 4) was conducted in an ICU setting while the remaining were conducted in isolation wards. In the same line, there was one study (Reference 5) that had categorized patients based on abnormal liver test (Group A) and liver injury (Group B). All articles had a NOS score of six and above. This meant that they were all of high quality.

**Table 1:** Description of liver dysfunction between survivors and non-survivors among included studies

Study, year	Setting	Country	Sample (dead/alive)	Age (dead/alive)	Chronic liver disease (dead/alive)	ALT (dead/alive)	AST (dead/alive)	Female gender	NOS
Yang et al. [7], 2020	ICU	China	32/20	64.6 (11.2)/51.9 (12.9)	NA	NA	NA	11/6	7
Wang et al. [13], 2020	Isolation ward	China	65/274	76 (70–83)/68 (64–74)	1/1	24 (19–49)/28 (17–43)	43 (30–68)/29 (22–43)	26/147	7
Chen N et al. [14], 2020	Isolation ward	China	113/161	68.0(62.0–77.0)/51.0(37.0–66.0)	5/6 (HBsAg +)	28.0 (18.0–47.0)/ 20.0 (14.8–32.0)	45.0 (31.0–67.0)/ 25.0 (20.0–33.3)	30/73	8
Zhou et al. [16] 2020	Isolation ward	China	54/135	69·0 (63·0–76·0)/52·0 (45·0–58·0)	NA	40.0 (24.0–51.0)/27.0 (15.0–40.0)	NA	16/56	7
Du et al. [15], 2020	Isolation ward	China	21/158	70.2(7.7)/56.0(13.5)	2/2	27.0 (20.0‒37.0)/22.0 (14.0‒40.5)	40.0 (27.0‒61.5)/27.5 (19.0‒42.0)	11/71	7

Data expressed by mean (SD) or median (IQR), *IQR* interquartile range, *ICU* intensive care unit, *ALT* alanine aminotransferase (> 41 U/L), *AST* aspartate aminotransferase (> 40 U/L), *HBsAg* Hepatitis B virus surface antigen positivity

**Table 2:** Description of liver dysfunction between non-severe and severe among included studies

Study, year	Setting	Country	Sample (non-severe/severe)	Age (non-severe/severe)	Chronic liver disease (non-severe/severe)	ALT (non-severe/severe)	AST (non-severe/severe)	TBIL. (non-severe/severe)	Female	NOS	
Chen guang et al. [^17^], 2020^a^	Isolation ward	China	10/11	52.0(42.8–56.0)/61.0 (56.5–66.0)	NA	16.0 (13.3–21.8)/42.0 (32.5–50.0)	24.0(21.5–26.5)/47.0 (28.0–74.5)	7.8 (6.4–9.5)/8.8 (7.9–10.5)	1/3	7	
Zheng et a. [^18^], 2020	Isolation ward	China	131/30	40 (31–51)/57 (46.5–66)	4/0	19.3 (14.6–17.8)/23.9 (17.6–35.3)	23.4 (19.0–28.8)/31.6 (25.9–49.36)	10.7 (8.18–15.3)/12.7 (9.2–16.9)	65/16	7	
Wan et al. [^11^], 2020	Isolation ward	China	95/40	44(33–49)/56(52–73)	1/1	21.7(14.8–36.9)/26.6(14.5–33.3)	22.4(16.9–30.5)/33.6(25.7–44.2)	8.6(5.6–14)/9.8(7.8–15.6)	43/19	8	
Zhang et al. [^19^], 2020^b^	Isolation ward	China	84/31	43.96 ± 14.84/64.58 ± 13.26	3/2 (Hepatitis B)	21.22 ± 12.67/37.87 ± 32.17	24.39 ± 9.79/38.87 ± 22.55	10.27 ± 4.26/14.12 ± 6.37	55/11	7	
Guan et al. [^20^], 2020^c^	Isolation ward	China	926/173	45.0(34.0–57.0)/52.0 (40.0–65.0)	22/1 (Hepatitis B)	120/606 vs 38/135	112/615 vs 56/142	59/594 vs 17/128	386/73	8	
Ji et al. [^21^], 2020	Isolation ward	China	163/39	42.9 (32.6 - 51.8)/55.1 (43.7 -71.8)	42/34 (NAFLD) 5/2 (HBsAg)	82/163 vs 19/39	24/163 vs 10/39	13/163 vs 4/39	77/12	7	
Cai et al. [8]. 2020^d^	Isolation ward	China	233/85 (A) 47/43 (B)	NA	78/60 (A) 28/41 (B)	41 (23–65)/67 (47–100) (A) 84 (42–136)/96 (63–159) (B)	34(27–45)/58(41–93) (A) 45(29–81)/80(56–145) (B)	19 (13–26)/22 (18–28) (A) 22 (15–41)/25 (19–32) (B)	NA	7	
Li et al. [10], 2020^e^	Isolation ward	China	279/269	56 (44–66)/65 (54–72)	2/2	61/275 vs 64/266	64/275 vs 115/265	7/275 vs 17/266	153/116	7	

Data expressed by mean ± SD, median (IQR) or n/N. IQR = interquartile range. ALT: alanine aminotransferase (> 41 U/L). AST: aspartate aminotransferase (> 40 U/L). TBIL: total bilirubin (> 21 μmol/L). NAFLD: non-alcoholic fatty liver diseases. HBsAg: Hepatitis B virus surface antigen positivity

^a^Patients based on AST > 40U/L

^b^Patients based on ALT > 50U/L

^c^Patients based on TBIL > 17.1 μmol/L

^d^Patients based on abnormal liver test (Group A) and liver injury (Group B)

^e^Complication liver dysfunction included 44 non-severe and 62 severe patients

### Pooled analysis

#### Survivors vs non-survivors

**Fig. 2:**
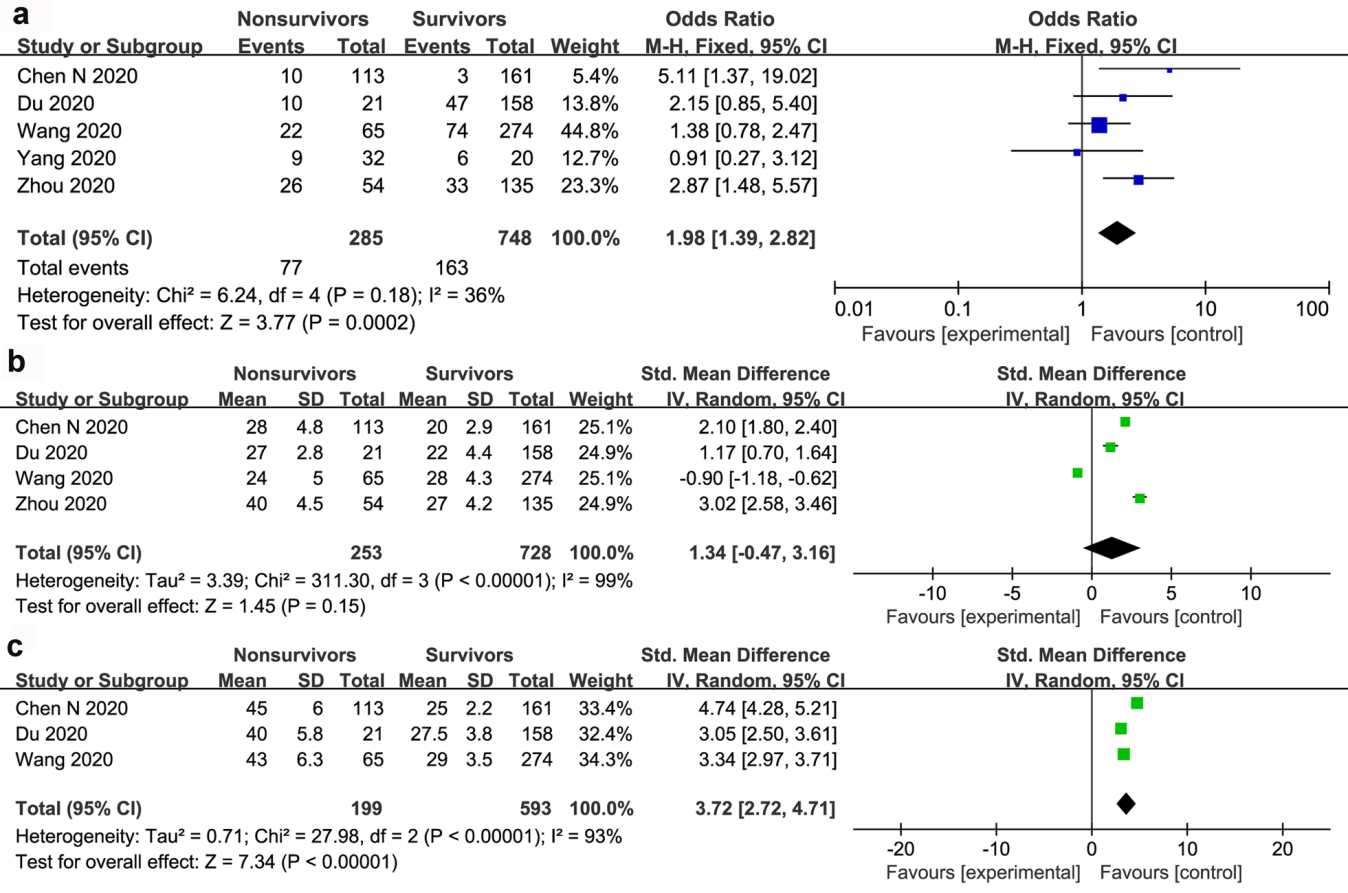
The forest plot outcome for the connection between liver dysfunction and the mortality of patients with COVID-19. **a** Events between non-survivors and survivors. **b** ALT levels between non-survivors and survivors. **c** AST levels between non-survivors and survivors

The forest plot outcome showing the association between liver dysfunction and the mortality of patients with COVID-19 is displayed in Fig. 2a. There was a significant connection between liver dysfunction and mortality of COVID-19 patients with a pooled OR of 1.98 (95% CI 1.39–2.82; *P* = 0.0002). The pooled data were calculated under the FEM as a low heterogeneity within the studies. Pooled results of five studies revealed that AST was significantly lower in COVID-19 survivors (WMD 3.72; 95% CI 2.74 to 4.71; *P* < 0.001) (Fig. 2c). However, there were no significant differences in ALT levels between COVID-19 survivors and non-survivors (WMD 1.34; 95% CI -0.47 to 3.16; *P* = 0.15) (Fig. 2b).

#### *Non-severe vs severe based on ALT*, *AST and TBIL*

**Fig. 3:**
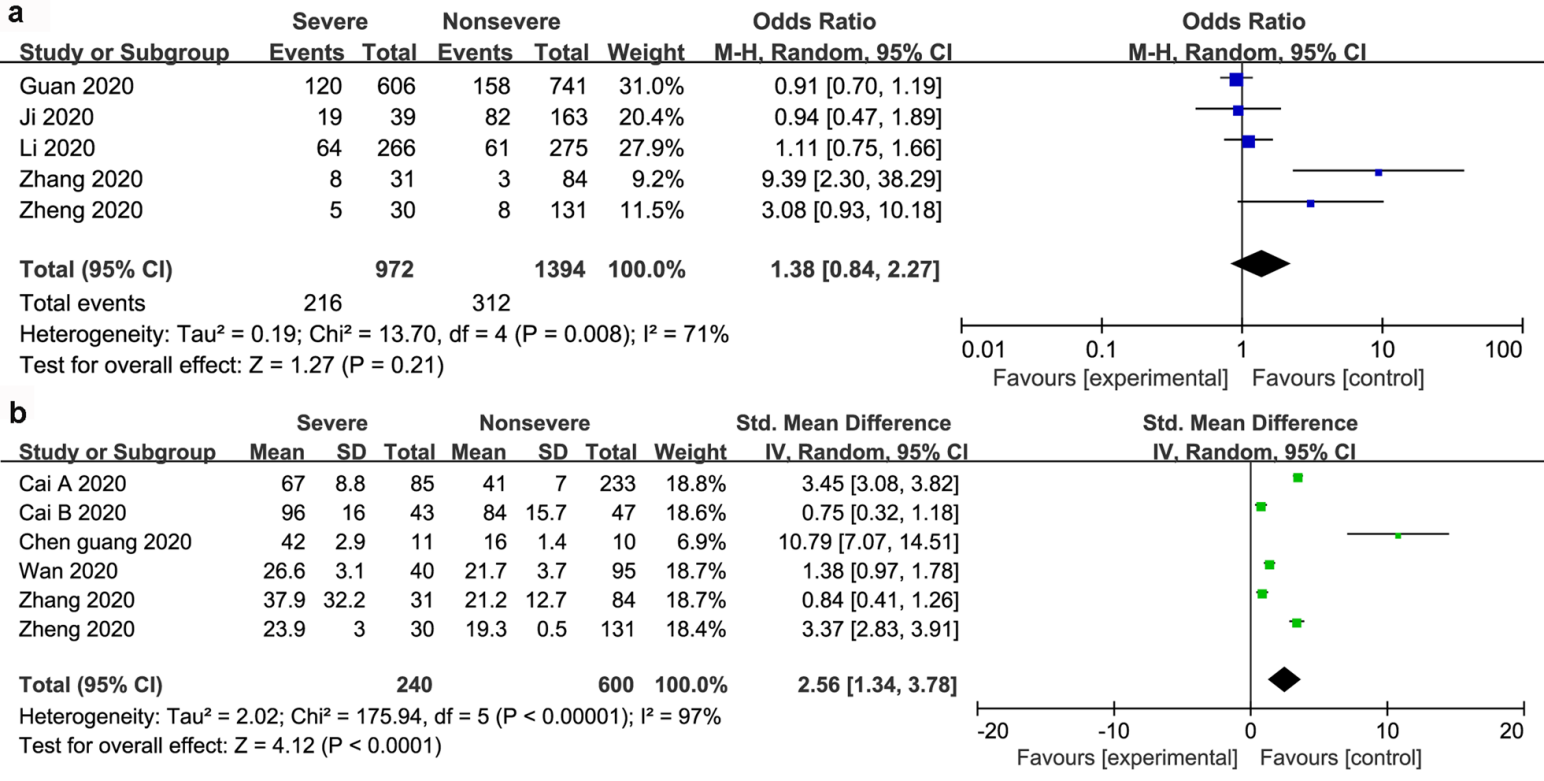
The forest plot outcome for the connection between ALT and the severity of patients with COVID-19. **a** Events between non-severe and severe patients. **b** ALT levels between non-severe and severe patients

**Fig. 4:**
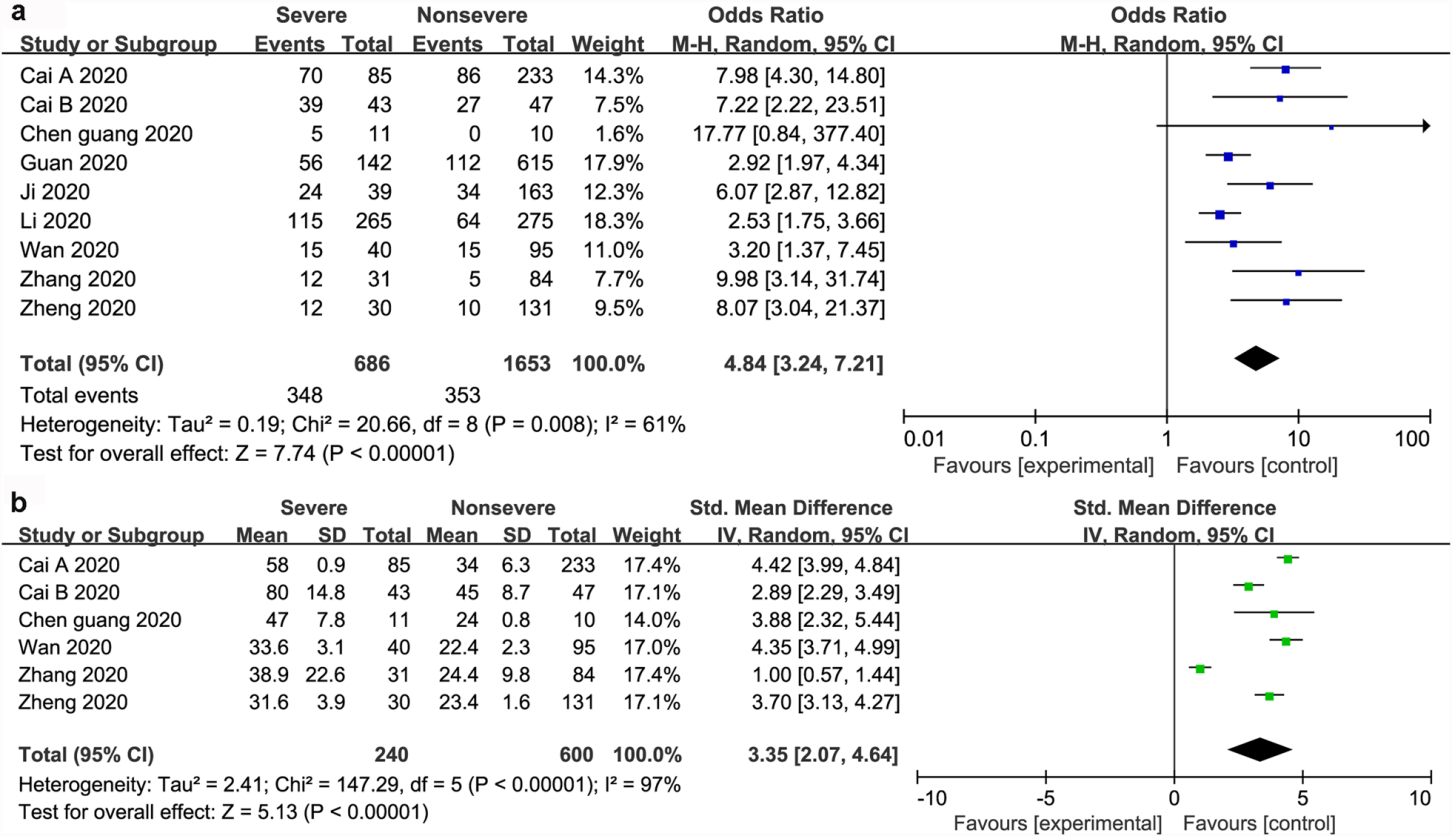
The forest plot outcome for the connection between AST and the severity of patients with COVID-19. **a** Events between non-severe and severe patients. **b** AST levels between non-severe and severe patients

**Fig. 5:**
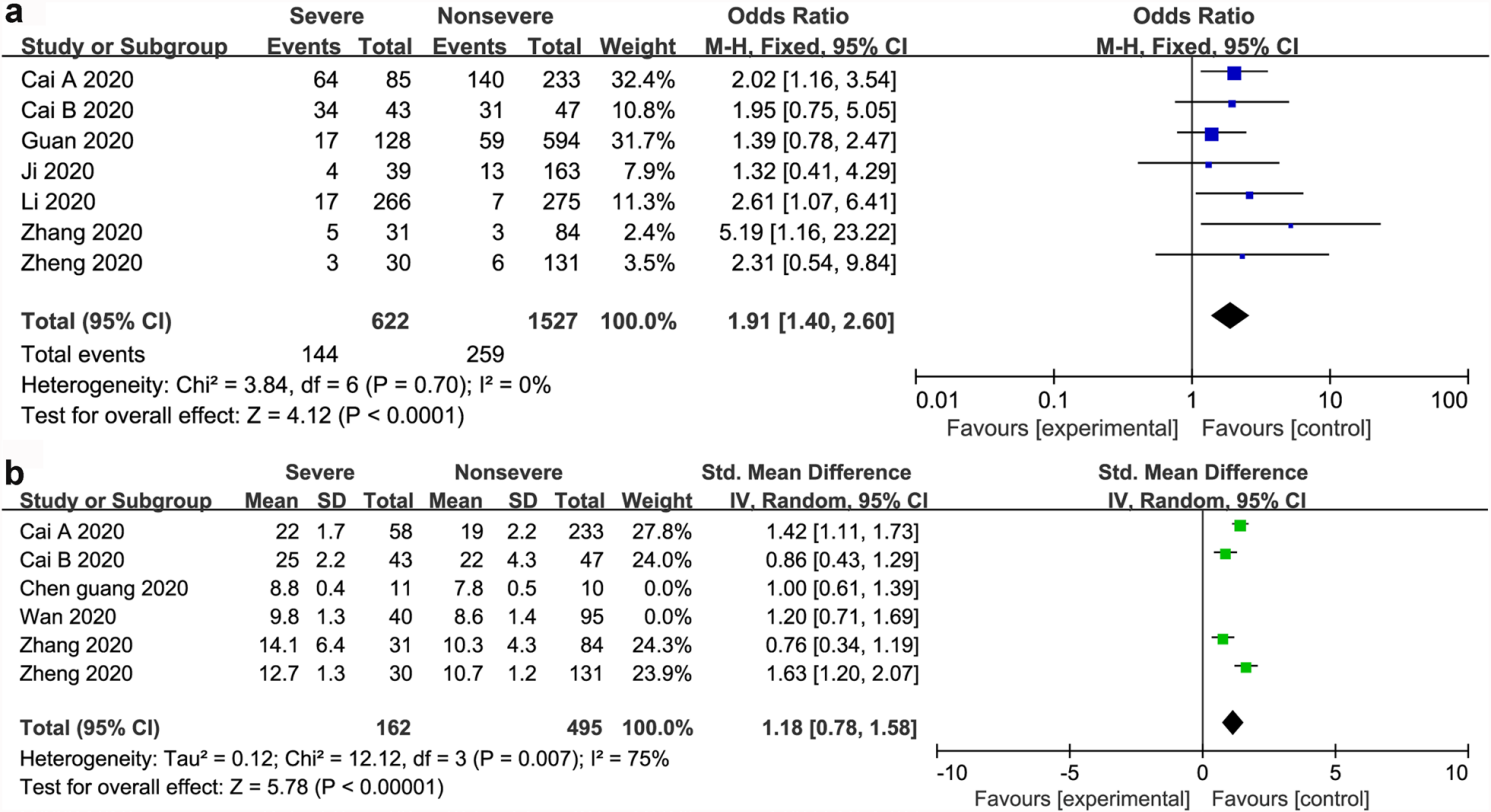
The forest plot outcome for the connection between TBIL and the severity of patients with COVID-19. **a** Events between non-severe and severe patients. **b** TBIL levels between non-severe and severe patients

Dichotomous and continuous analysis revealed that there was no significant association between ALT and severity of COVID-19 with a pooled OR of 1.38 (95% CI 0.84–2.27; *P* = 21). However, ALT was significantly lower in patients with non-severe COVID-19 (WMD 2.56; 95% CI 1.34 to 3.78; *P* < 0.001) (Fig. 3). In the same line, there was a significant association between AST and severity of COVID-19 with a pooled OR of 4.48 (95% CI 3.24–7.21; *P* < 0.001), and a pooled WMD of 3.35 (95% CI, 2.07 to 4.64; *P* < 0.001) (Fig. 4). Similarly, there was a significant association between TBIL and severity of COVID-19 with a pooled OR of 1.91 (95% CI 1.40–2.60; *P* < 0.001), and a pooled WMD of 1.18 (95% CI 0.78 to 1.58; *P* < 0.001) (Fig. 5).

#### Subgroup analysis and sensitivity analysis

Subgroup analysis was used to reveal the sources of heterogeneity. Age and the number of non-severe patients were found to be potential sources of heterogeneity (Table 3). However, the impact of age stratification should be verified further using bigger data sets because this study had a small data set. The overall mortality of COVID-19 had low heterogeneity (*I*^*2*^ = 36%). However, when either Chen’s or Wang’s study was eliminated, the results significantly affect the pooled outcomes (*I*^*2*^ dropped from 36 to 25%). Similarly, when Chen’s study was removed from the AST group, the *I*^*2*^ dropped from 93 to 70%. In the same line, the severity of COVID-19 in AST (events) had moderate heterogeneity (*I*^*2*^ = 61%). However, when Li’s study was eliminated, the *I*^*2*^ dropped from 61 to 49%. Similarly, when Zhang’s study was removed in the AST (levels) group, the *I*^*2*^ dropped from 97 to 79%. When Zhang’s study was removed in the AST (events) group, the *I*^*2*^ dropped from 71 to 28%. In the TBIL (levels) group, removal of Zheng’s study led to reduction of the *I*^*2*^ from 71 to 67%. Cognizant of this, it was hypothesized that the studies of Zhang et al*.* 2020 and Zheng et al*.* 2020 were the sources of heterogeneity in this meta-analysis. The Egger^’^s regression test (*P* > 0.05) further revealed that the meta-analysis had no significant publication bias.

**Table 3:** Results of subgroup analysis among included studies

Subgroup	Studies sample size	Included (N) (study/control)	Chi-square (*df*)	*P* value	Pooled overall heterogeneity	SMD (95% CI) (*I*^*2*^)
Non-survivors vs survivors ALS (levels)						
Age≥70	2	86/432	0.72 (1)	.001	3.25 (2.95-3.56)	0
Severe vs non-severe ALT (levels)						
Number of non-severe≥100	2	115/364	0.06 (1)	.001	3.42 (3.12-3.73)	0
Severe vs non-severe AST (events)						
Age≥60	6	379/1284	11.17 (5)	.001	5.09 (3.26-7.94)	55
Severe vs non-severe AST (levels)						
Age≥60	3	81/236	2.21 (2)	.001	3.98 (3.54-4.43)	10
Severe vs non-severe TBIL (levels)						
Number of non-severe≥100	2	88/364	0.62 (1)	.001	1.49 (1.24-1.74)	0
Number of non-severe<100	4	125/236	5.14 (3)	.001	0.93 (0.61-1.25)	42

## Discussion

Herein, the abnormal liver function was associated with mortality and severity of COVID-19. Subgroup analysis conducted to identify sources of heterogeneity further revealed that the overall outcome was acceptable. Similarly, sensitivity analysis revealed that when any study was excluded or REM was converted to FEM, the overall outcome was acceptable. These results strongly suggested that there in a correlation between liver injury and COVID-19.

Although it has been observed that the most severe and fatal cases of COVID-19 occur in the elderly individuals with liver injury, the relevant pathophysiology is still unclear. Xu et al [22] reported the pathological characteristics of the liver of a 50-year-old man who died from critical COVID-19. His liver had moderate microvascular steatosis as well as mild lobular and portal activity. However, no histological features of liver failure and bile duct injuries have been observed until now. SARS-CoV-2 is closely related to SARS-CoV by virtue of both sharing the same receptor (ACE2). However, there is no relevant study that has explored the role of ACE2 in liver injury especially in COVID-19 patients given that majority of them usually have elevated serum aminotransferase. The over-activated immune response caused by SARS-CoV-2 infection and systemic inflammation associated with cytokine storms may lead to organ (liver) damage [23]. Qin et al*. *[24] reported that most severe COVID-19 patients had elevated biomarkers associated with infection, increased inflammatory cytokine levels, and decreased number of T cells. In addition, stress (such as shock, ARDS, and septic) or drug toxicity-induced liver injury might be associated with hypoxia reoxygenation, oxidative stress, and imbalance of acid–base. This also contributes to very high aminotransferase concentrations. Nonetheless, studies have reported varying levels of aminotransferase. This meta-analysis revealed that there is a significant correlation between COVID-19 and liver dysfunction which provides a basis for better clinical management of COVID-19 and liver disease patients.

Patients aged above 65 years have higher comorbidities, more severe symptoms, and are more prone to multiple organ involvement and death compared to the younger patients [13]. For example, Feng et al*.* 2020 reported that the survival rate of patients aged above 75 years was significantly lower than that of young patients [25]. Factors leading to poor health outcomes include physiological changes in aging and various age-related complications. Moreover, older people's suspicion and detection thresholds for SARS-2 such as temperature, decreased function of cough, and shortness of breath are lower [26]. Cognizant of this, more attention should be paid to COVID-19 patients with a history of liver disease (especially elderly patients) by constantly observing liver changes, and carefully determining the cause of liver abnormalities. Age subgroup analysis revealed that the age ≥ 70 or age ≥ 60 subgroup is of great significance. However, further studies should be conducted on the effect of age stratification on the mortality and severity of patients with COVID-19 combined with liver injury.

Previous studies quantify the effects of COVID-19 on the digestive system and chronic liver disease [27, 28]. While this meta-analysis to prove that there is an association between severity and mortality of COVID-19 patients, and liver dysfunction. Nevertheless, this study was limited by several factors. Additional information such as liver disease types, drug use, nutritional levels, and other indicators to assess liver function was not present. As such, stratifying the risk in subgroup analysis of liver injury patient populations was not possible. In the same line, only the information regarding the age and gender of COVID-19 patients was collected. Other factors such as BMI, measurement methods and instruments for detecting SARS-CoV-2, and sample size could have as well affected the accuracy of the results. Moreover, patients diagnosed with COVID-19 could have had multiple chronic diseases such as intestinal diseases and pre-existing liver disease that could have affected the accuracy of the results. As such, large-scale prospective studies should be conducted to verify these results.

## Conclusion

The mortality and severity of COVID-19 patients are significantly associated with liver dysfunction. The non-survivors and severe COVID-19 patients have elevated serum AST levels than the survivors and non-severe COVID-19 patients. The results of this study form a basis for better clinical liver management of patients with COVID-19.

## Data Availability

Data sharing is not applicable to this article as no datasets were generated or analyzed during the current study.

## Abbreviations

ACE2: Angiotensin converting enzyme 2
SARS-CoV-2: Severe acute respiratory syndrome coronavirus 2
COV: Coronaviruses
AST: Aspartate aminotransferase
ALT: Serum alanine aminotransferase
REM: Random effect model
FEM: Fixed effect model
OR: Odds ratio
WMD: Weighted mean difference
CI: Confidence intervals
ICU: Intensive care unit
TBIL: Total bilirubin
